# In Vitro Antibody Quantification with Hyperspectral Imaging in a Large Field of View for Clinical Applications

**DOI:** 10.3390/bioengineering10030370

**Published:** 2023-03-17

**Authors:** Martina De Landro, Lorenzo Cinelli, Nicola Marchese, Giulia Spano, Manuel Barberio, Cindy Vincent, Jacques Marescaux, Didier Mutter, Michel De Mathelin, Sylvain Gioux, Eric Felli, Paola Saccomandi, Michele Diana

**Affiliations:** 1 Department of Mechanical Engineering, Politecnico di Milano, 20156 Milan, Italy; 2 Department of Gastrointestinal Surgery, San Raffaele Hospital IRCCS, 20127 Milan, Italy; 3 Research Institute against Digestive Cancer (IRCAD), 67000 Strasbourg, France; 4 Department of General Surgery, Ospedale Card. G. Panico, 73039 Tricase, Italy; 5 Institut de Chirurgie Guidéè par L’image, University Hospital Institute (IHU), 67000 Strasbourg, France; 6 Digestive and Endocrine Surgery, Nouvel Hopital Civil, University of Strasbourg, 67000 Strasbourg, France; 7 ICube Laboratory, Photonics Instrumentation for Health, 67400 Strasbourg, France; 8 Intuitive Surgical, 1170 Aubonne, Switzerland

**Keywords:** hyperspectral imaging, antibody quantification, intraoperative sensing, tumor target detection, HMGB1

## Abstract

Hyperspectral imaging (HSI) is a non-invasive, contrast-free optical-based tool that has recently been applied in medical and basic research fields. The opportunity to use HSI to identify exogenous tumor markers in a large field of view (LFOV) could increase precision in oncological diagnosis and surgical treatment. In this study, the anti-high mobility group B1 (HMGB1) labeled with Alexa fluorophore (647 nm) was used as the target molecule. This is the proof-of-concept of HSI’s ability to quantify antibodies via an in vitro setting. A first test was performed to understand whether the relative absorbance provided by the HSI camera was dependent on volume at a 1:1 concentration. A serial dilution of 1:1, 10, 100, 1000, and 10,000 with phosphatase-buffered saline (PBS) was then used to test the sensitivity of the camera at the minimum and maximum volumes. For the analysis, images at 640 nm were extracted from the hypercubes according to peak signals matching the specificities of the antibody manufacturer. The results showed a positive correlation between relative absorbance and volume (r = 0.9709, *p* = 0.0013). The correlation between concentration and relative absorbance at min (1 µL) and max (20 µL) volume showed r = 0.9925, *p* < 0.0001, and r = 0.9992, *p* < 0.0001, respectively. These results demonstrate the HSI potential in quantifying HMGB1, hence deserving further studies in ex vivo and in vivo settings.

## 1. Introduction

In the last decade, a new vision-based modality has emerged in the biomedical field: hyperspectral imaging (HSI). This optical imaging modality allows for measuring spectral information from across the electromagnetic spectrum. For medical applications, the most interesting and investigated spectral range spans the wavelengths between the visible and near-infrared range (e.g., from 450 to 1000 nm). HSI offers great potential in both diagnostic and therapeutic areas. Therefore, this innovative spectral imaging technology can find potential applications in pathology, histology, and clinical diagnosis. Furthermore, it is a promising optical tool for surgical guidance [1]. Recently, researchers have used microscopic hyperspectral technology to identify color and texture differences, classify label-free pathological tissue, and implement carcinoma diagnosis with deep-learning techniques [2,3]. It is indeed known that the way a biological structure or a tissue interacts with light can reveal the current status of the structure, such as the progression of the disease or the effect of a systemic localized therapy.

HSI relies on the autofluorescence of the tissue chromophores to quantify and spatially resolve the relative absorbance of endogenous organic compounds, such as oxygenated and deoxygenated hemoglobin and antibodies, in a wide wavelength range, providing intraoperative evaluation in the large field of view (LFOV) [4]. When interacting with light, a biological structure undergoes several phenomena, such as absorption and scattering. Absorption is due to the natural presence of chromophores in the tissue, such as hemoglobin and water, whereas scattering is caused by the inhomogeneous structure [5]. Therefore, the light reflected and transmitted from the medium carries quantitative diagnostic information about the medium’s state and content.

HSI is a non-invasive, contrast-free tool recently applied in several medical and basic research fields [5,6], like other new emerging technologies [7,8]. Several innovative approaches are under investigation that exploit the capability of HSI to “see beyond” the visible range, such as intraoperative tissue recognition [9], therapy guidance, and monitoring [10,11,12,13,14]. 

Another important field of application of HSI is cancer detection and in situ biopsy [15,16], along with the identification of tumor boundaries for surgery guidance [17]. Today, microscopic (R1) or gross (R2) tumor involvement at surgical resection margins represents the main predictor of local recurrence and shortens the survival rate in various cancers [18,19,20,21,22,23]. On the other hand, late-stage diagnosis remains one of the main factors responsible for treatment failure and a major issue for current screening programs. Data on anatomy are usually obtained thanks to ultrasonography (US), computed tomography (CT) scans, magnetic resonance imaging (MRI), and endoscopic techniques. The obtained information is fundamental for decision-making on respectability; however, these technologies lack the high spatial and marker-specific accuracy required to guarantee precision in the identification, leading to reduced effectiveness in using pre-operative images for intra-operative surgical guidance. Moreover, the current lack of commercial imaging systems that can provide an intraoperative quantification of labeled antibodies (Ab) for low-in-depth cancerous lesions makes hyperspectral imaging (HSI) one of the most interesting and clinically transferable tools to test.

Currently, the final decision-making on resection margins depends on intraoperative evaluation based on visual appearance, palpation, and frozen section analysis. Despite high accuracy, frozen section examination results are time-consuming and limited by the number of potential samples, artifacts related to surgical devices, and the pathologist’s specialization [24,25]. The gap between preoperative imaging assessment and surgical findings could be reduced by image-guided surgery, providing in situ and real-time visualization of the tumor through new optical systems [26]. 

In this work, we investigate the performance of an HSI system to detect and quantify an antibody (high mobility group box 1 (HMGB1)) in a laboratory setting. We selected HMGB1 because it is a ubiquitous nuclear protein involved in maintaining genomic structure and function in almost all eukaryotic cells [27]. A recent review stated the role of HMGB1 in orchestrating tumorigenesis and inflammation-related immunosuppression, resulting in overexpression in neoplastic cells of various types of tumors [28]. According to such assumptions, HMGB1 could be used as a specific target for many human cancers. HMGB1 was labeled with Alexa fluorophore (647 nm) to reproduce a chromophore that could be employed in future ex vivo and in vivo preclinical applications and that was never tested before.

## 2. Materials and Methods

### 2.1. Sampling

**Figure 1:**
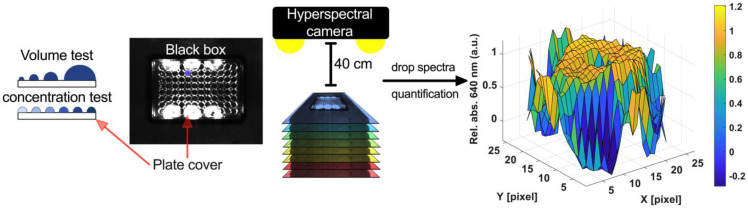
The relative absorbance of the drops (labeled antibody dissolved in PBS) was collected with the HSI camera and used to quantify Ab. Sampling drops at different volumes and concentrations were placed on the cover of a transparent 96-well plate in a black box at a 40 cm distance from the Hyperspectral camera. The distance (40 cm) is set up by the manufacturer to guarantee LFOV during the surgical procedure.

The experimental workflow of the study is reported in Figure 1. A first test was performed to understand if relative absorbance was dependent upon volume at a 1:1 concentration. A serial dilution of 1:1, 10, 100, 1000, 10,000 with phosphatase-buffered saline (PBS) was then used to test the sensitivity of the camera at the minimum and maximum volumes tested in the first phase. To perform the test, the drop was placed over a transparent cover of a 96-well plate in a black box to optimize the signal from the object in the foreground obtained with the HSI camera. For the analysis, images at 640 nm were extracted from the hypercubes according to peak signals matching the specificities of the antibody manufacturer. Finally, the relative absorbance in the pixel of interest was analyzed and correlated with the volume and concentration. 

### 2.2. Hyperspectral Imaging

Hyperspectral images were generated using a CMOS push-broom scanning hyperspectral camera (TIVITA, Diaspective Vision GmbH, Am Salzhaff-Pepelow, Germany). The acquisition of a single hypercube is performed with a camera-specific module of the Perception Studio software (Perception Park 1.7, Graz, Austria) within 6 s in every sampling. The Ab was excited with light emitted by a 20 W Osram Halospot 70 Halogen lamp (6×). Final hypercubes have dimensions of 640 by 480 by 100 (*X* × *Y* × *wavelength*). The spectral range of this camera is 500–995 nm. The LFOV was granted by the distance between the objective of the camera and the drop, which was 40 cm. Therefore, the final field of view (FOW) included the entire 96-well plate, thus providing spectral information at multiple points. To maintain the same distance in each sample, we measured the distance at every sampling with a Bluefruit Feather nRF52832 with Adafruit VL53LOx device. The wavelength was calibrated using defined emission peaks of a krypton gas lamp during production. Meanwhile, the noise of the sensor was corrected for time and temperature effects. Specifically, dark current effects and sensor noise patterns are recorded and corrected by the developed software component. To convert image data from radiance to relative reflectance, a white reference object characterized by a high diffuse reflectance is used to create a reference cube before the measurements start. Relative reflectance was extracted and then converted into the relative absorbance with the following formula: $A=-\ln\left(\frac{I}{I_{0}}\right).$ Additional details about camera calibration, balancing, and extraction of the final hypercube were previously discussed by Holmer et al. in 2008 [29].

### 2.3. Antibody

For this study, an anti-HMGB1 monoclonal antibody (Alexa Fluor 647, BioLegend, Basel, Switzerland) was used. Its peak of absorbance is at 647 nm and its emission is at 668 nm when it is excited at 633/635 nm as per the manufacturer’s instructions. The dilution of the antibody was performed with PBS (Phosphatase-Buffer Solution, Gibco, Basel, Switzerland).

### 2.4. Statistics

Statistics were performed with GraphPad 8.3 (Prism, GraphPad Software, San Diego, CA, USA). One-way ANOVA, Brown-Forsythe with Welch test, and two-way ANOVA were performed depending on the assumptions for continuous values. Spearman’s and Pearson’s correlation were used for the correlation analysis. A two-tailed analysis with a *p*-value < 0.05 was considered statistically significant. Three-dimensional graphs were performed with MATLAB 2020a. 

## 3. Results

**Figure 2:**
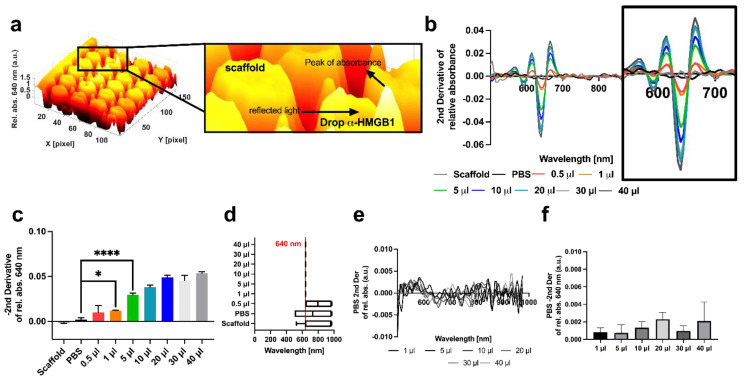
HSI Ab detection and volume size tests were performed as follows. (**a**) A drop of antibody 1:1 (antibody/PBS) was placed over the cover of a 96-well plate and HSI was performed. (**b**) Second derivative of relative absorbance in the selected ROI where the drops were placed. The analysis of the peak indicated a peak of absorbance at 640 nm after 1 mL. Each signal is an average of 4 drops. (**c**) The second derivative of relative absorbance showed peaks at 640 nm proportional to volume. The maximum significant increase was found at 5 mL (*p* < 0.0001), after which the *p*-value did not change. (**d**) The statistical analysis showing the minimum volume detectable was 0.5 mL, where a signal/volume plateau phase was found after 20 mL (7 different volumes were tested). (**e**,**f**) The PBS volume/signal test showed that the PBS volume did not significantly contribute to the signal at 640 nm. Data are presented as mean +/− s.d. and compared to the scaffold with one-way ANOVA, ns *p* > 0.05, * *p* ≤ 0.05, **** *p* ≤ 0.0001. N = 4 drops per volume type. Spectra were displayed by using the second derivative to show how the peak was detected over the entire range (500–995 nm). The volume corresponding to the minimum and maximum significant signals (1 mL and 20 mL, respectively) over the PBS was further analyzed. At 1 mL, the 1:4 dilution (0.0125 mg/mL) was the first one which was found to be significantly different as compared to the PBS (0.0046 ± 0.0014 1:4 and 0.0017 ± 0.0019 PBS respectively, *p* = 0.0032) (Figure 3a,b). The peak contribution at 640 nm presented a distributed maximum value on multiple wavelengths when compared to 1:2 (0.0251 mg/mL) (Figure 3c). The dilution at 1:2 was statistically different from the PBS (0.0112 ± 0.0015, *p* < 0.0001). The dilution test performed with 20 mL drops showed a significant signal at 1:16 (0.0031 mg/mL) (0.0035 ± 0.0009, *p* = 0.0228) (Figure 3d,e). The peak test analysis showed that the minimum dilution with a sharp peak was 1:8 (0.0062 mg/mL) (Figure 3f), which was statistically significant (0.0062 ± 0.0008, *p* < 0.0001).

To visualize the signal of relative absorbance over the scaffold, the HSI image at 640 nm was plotted in Figure 2a. The second derivative of the relative absorbance was extracted from each hypercube using the TIVITA software, and different peaks were found proportionally to volume size (Figure 2b). The region of interest (ROI) of the drop was selected, and the spectral profile was extracted as the negative second derivative of the relative absorbance. The drop was placed in the center of every well on the cover which was well delimited by the manufacturer. The entire spectrum (500–995 nm) was analyzed to check the minimum volume detected at 640 nm and to find the plateau phase on the volume/signal relation. The minimum significant signal was found at 1 mL (0.0119 ± 0.0006 a.u., *p* = 0.0145) when compared to the PBS (0.0021 ± 0.0022 a.u.) (Figure 2c). The threshold was observed at 20 mL (0.0490 ± 0.0025 a.u.), after which no significant increase in signal was found. The negative peak was isolated in each volume; the average of observations was consistent at 640 nm between 1 mL and 40 mL; the scaffold, PBS and 0.5 mL showed not specific antibody peak (Figure 2d). The PBS absorbance at 640 nm was tested from a 1 mL to a 20 mL volume, revealing that its contribution did not significantly increase with volume (Figure 2e,f). Additionally, the contribution of PBS to signal was compared to PBS mixed with the antibody, showing a minimal-to-absent contribution to an overall absorbance at 640 nm (Figure S1 in Supplementary Materials). 

## 4. Discussion

HSI is a novel optical tool recently applied in medical and basic research fields. By providing spectral and spatial information on the object of interest, HSI can measure the relative absorbance of endogenous organic compounds pixel by pixel in large areas of interest. In this work, the accuracy of HSI in detecting exogenous molecules in an LFOV has been preliminarily explored in vitro, showing promising results. The ability to quantify and localize intraoperative molecular changes in the tissue could be useful in providing molecular-guided surgery, leading to a more precise surgical intervention.

Several biological molecules have been studied during the last two decades to discriminate between healthy and tumoral tissues. However, few articles explored their potential for intraoperative navigation. 

Sano et al. [30] described using the NIR-labeled human anti-epidermal growth factor receptor (EGFR) (panitumumab) and indocyanine green (ICG) conjugated with human anti-epidermal growth factor receptor type 2 (HER2) (trastuzumab) to obtain a multi-color target-specific fluorescence breast cancer imaging in an in vivo mouse model. However, it required two fluorescent dyes, and HER2 is overexpressed only in 15 to 30% of all invasive breast cancers [31]. A randomized controlled multicentric phase III trial by Stummer et al. [32] considered 5-aminolevulinic acids (5-ALA) as a tracer dye, leading to the accumulation of fluorescent porphyrins within malignant glioma tissue. However, this study did not provide preoperative molecular or genetic information regarding 5-ALA susceptibility to brain tumors. 

Using HSI to detect Ab against HMGB1 has the potential to overcome the limitations of the abovementioned techniques. HSI could discriminate between the PBS from the scaffold and the antibody drops placed on the surface of the 96-well plate cover. The minimum concentrations detected for 1 mL were 0.0125 mg/mL and 0.0031 mg/mL. A high positive significant correlation was found between volume and concentration with a relative absorbance at 640 nm. These findings suggest considering the identification of HMGB1 performed through HSI as a useful tool for image-guided surgery, but also for a preoperative malignant diagnosis. 

The results of our study confirm that spectral imaging is a powerful technology that can be used to detect biological changes that cannot be identified with traditional gray or color imaging methods. Unlike traditional optical diagnostic methods, HSI technology can collect the entire spectrum for each pixel in the image over a selected wavelength range, providing a more consistent spatial/spectral-based analysis. This technology opens new prospects in biology and medicine. However, there is no unified standard to make these systems easily compatible and comparable. This aspect may strongly limit its spread. Additionally, the biomedical spectral imaging analysis mechanism is not so clear [2]. Several studies are underway to better understand the spectrum-physiological link. 

It has already been proven that Ab is a suitable tool for the preoperative endoscopic detection of malignancies. In 2017, Nagengast et al. [33] presented a study on the identification of esophageal adenocarcinoma and dysplasia in a selected population affected by Barrett’s esophagus, a complex condition requiring periodic endoscopic examinations with serial biopsies. They used NIR fluorescence molecular endoscopy (NIR-FME) after systemic and topical administration of a fluorescence-labeled antibody against the vascular endothelial growth factor (VEGFA) (bevacizumab 800CW) to guide endoscopic mucosal resection. The topical administration of the tracer consisted in spraying bevacizumab 800CW on the mucosal surface (100 μg/mL per cm of Barrett’s esophagus) immediately before NIR-FME. As a result, the topical application showed a 25% higher detection rate when compared to white light endoscopy and the systemic administration of dye, showing more accuracy and precision in the detection of dysplastic and neoplastic lesions. However, this study included a small population, with a selection bias due to the choice of patients already scheduled to undergo endoscopic mucosal resection of previously diagnosed lesions. In a challenging situation such as Barrett’s esophagus, the HSI detection of Ab has the potential to provide identification of dysplastic or malignant cells during screening endoscopy. 

HSI has also demonstrated abilities to directly detect tumors. In that case, intra- and inter-patient variability in the spectral signatures of the same tissue can be a limitation, together with the challenge of scanning biological tissues. Additionally, according to the specific tumor target, different parts of the spectra may be somewhat significant [15,34,35]. Therefore, time acquisition may be a drawback, as well as the necessity to adjust the spectral range of interest for each tumor. Using a specific exogenous tumor marker overcomes such limitations, providing one single wavelength-based analysis. According to the abovementioned literature, the results of the present in vitro experiment suggested that the topical application of Alexa Fluor 647 on the esophageal mucosa could be a suitable and reproducible method. Moreover, since HMGB1 is overexpressed in both squamous cell carcinoma [36] and esophageal adenocarcinoma [37], it could be used to extend the analysis to all esophageal tracts. 

Our study experiment has several limitations. First, HSI has not yet been adapted to minimally invasive and endoscopic systems, and it still requires proper illumination, which often causes artifacts in the images. Secondly, in its current form, the video rate is not yet available, although an enhanced reality approach has already been developed by our team [38,39]. Additionally, due to the spectral resolution of the HS camera, we selected 640 nm as a suboptimal approximation of the Alexa fluorophore peak (647 nm). This could play a key role in errors generated in margin detection, especially in in vivo experiments. Additionally, the signal in the ROI containing the entire drop was considered as the information of interest. 

In conclusion, in the context of antibody quantification for performing molecular-guided surgery, relative absorbance must be provided in real-time, thus demanding short acquisition and data processing time. Additionally, the spectral interval should fit the wavelengths of interest following the specific antibody, with consistent spectral resolution around the selected value. Microscopic hyperspectral imaging, providing quite high spatial resolution, may also enhance the final abilities of the camera in antibody detection. 

As a result, the final limitation of the present study was the experimental setting itself, since the HSI accuracy could be affected using biological tissue and in vivo conditions. Type of organ, tissue optical properties and subsequent intrinsic tissue scattering and absorption, preoperative treatments, preexisting clinical and pathological conditions (e.g., Barrett’s esophagus), and intraoperative movement artifacts or blood perfusion are only a few of the overall variables which could impair the ability of HSI to detect tumor margins.

## 5. Conclusions

This study demonstrated the ability of HSI technology to determine the concentration of HMGB1 in LFOV. This work, conducted in vitro, showed a significant correlation between the relative absorbance at 640 nm of the antibody with sample volume and concentration. These findings suggest the his-based detection of HMGB1 could be a useful tool for medical applications such as image-guided surgery and preoperative malignant diagnosis. Nevertheless, this ability needs to be explored in ex vivo and in vivo settings to understand how the signal could be affected in such scenarios.

## Figures and Tables

**Figure 3:**
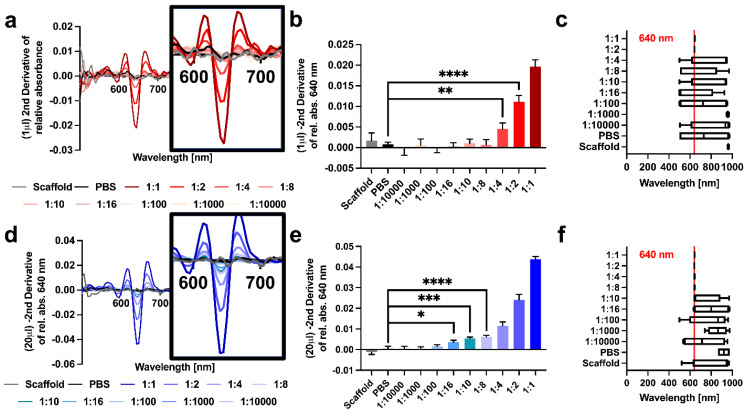
HSI Ab detection, concentration test at 1 mL and 20 mL. (**a**) The second derivative of relative absorbance calculated within the selected ROI in correspondence to each drop. Each signal is the average of 4 drops (N = 4) (1 mL) (min volume). (**b**) Negative second derivative of the 640 nm signal extracted from the hypercube at a 1 mL volume. (**c**) Peak test analysis where each drop peak was isolated (1 mL) (min volume). (**d**) Second derivative of relative absorbance calculated within the selected ROI in correspondence with each drop. Each signal is the average of 4 drops (20 mL) (max volume). (**e**) Negative second derivative of the 640 nm signal extracted from the hypercube at a 20 mL volume (max volume). (**f**) Peak test analysis where each drop peak was isolated (20 mL). Data are presented as mean +/− s.d. and compared to the scaffold with one-way ANOVA, ns *p* > 0.05, * *p* ≤ 0.05, ** *p* ≤ 0.01, *** *p* ≤ 0.001, **** *p* ≤ 0.0001. N = 4 drops per concentration type (9 different concentrations were tested). A positive correlation between relative absorbance at 640 nm and volume was found (r = 0.9709, *p* = 0.0013); see Figure 4a. Data are expressed in logarithmic form. The correlation between concentration and relative absorbance at 640 nm was performed at minimum and maximum volumes (Figure 4b). Both volumes were significantly positively correlated (r = 0.9925, *p* < 0.0001 1 mL and r = 0.9992, *p* < 0.0001 20 mL, respectively). In particular, the relative absorbance exhibits a linear relationship with the HMGB1 concentration, at different volumes. Linear relationship was also found between the antibody weight (mg) and second derivative of relative absorbance at maximum volume (blue in Figure 4c) while performing concentration tests. On the other hand, the volume test revealed a non-linear relationship between weight and the second derivative of relative absorbance (red in Figure 4c).

**Figure 4:**
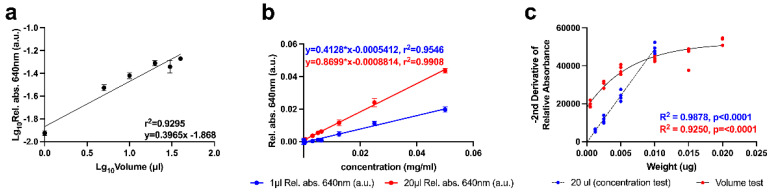
Correlation analysis was performed for (**a**) volume/relative absorbance at 640 nm (N = 6); (**b**) Concentration/relative absorbance at 1 mL (blue) and 20 mL (red). Pearson’s correlation test was performed; data are presented as mean +/− s.d. (N = 9 with 4 replicas for each measurement). (**c**) Correlation analysis for weight/second derivative of relative absorbance by varying concentration at 20 mL (in blue performed with Pearson’s correlation showing r = 0.9939) and volume (in red performed with Spearman showing r = 0.9587).

## Data Availability

The data presented in the study are available on request from the corresponding author.

## Supplementary Materials

The following supporting information can be downloaded at: https://www.mdpi.com/article/10.3390/bioengineering10030370/s1, Figure S1: Contribution of PBS to relative absorbance at 640 nm.
