# Supplementary material for: In Vitro Antibody Quantification with Hyperspectral Imaging in a Large Field of View for Clinical Applications
Source: Bioengineering (Basel). 2023 Mar 17;10(3):370. doi: 10.3390/bioengineering10030370 (PMC10045535; doi:10.3390/bioengineering10030370)
Supplement: Supplementary file 1 [file bioengineering-10-00370-s001.zip › bioengineering-2268567-supplementary.pdf]

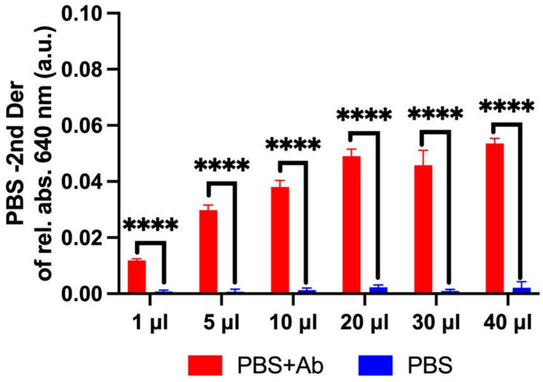

Figure S1. The second derivative of relative absorbance showed peaks at 640 nm at every volume tested. In every test, the antibody significantly contributed to the signal over the PBS ($p<0.0001$). Data are presented as mean +/- s.d. and compared to the scaffold with Two-way ANOVA with multiple T-test, ****$p \leq 0.0001$. N=4 drops per volume type. Spectra were displayed by using the 2nd derivative to show how the peak was detected over the entire range (500-995nm).
